# Home Literacy Activities and Children’s Reading Skills, Independent Reading, and Interest in Literacy Activities From Kindergarten to Grade 2

**DOI:** 10.3389/fpsyg.2020.01508

**Published:** 2020-07-02

**Authors:** Gintautas Silinskas, Monique Sénéchal, Minna Torppa, Marja-Kristiina Lerkkanen

**Affiliations:** ^1^Department of Psychology, University of Jyväskylä, Jyväskylä, Finland; ^2^Department of Psychology, Carleton University, Ottawa, ON, Canada; ^3^Department of Teacher Education, University of Jyväskylä, Jyväskylä, Finland

**Keywords:** home literacy activities, independent reading, early literacy, reading skills, kindergarten to Grade 2

## Abstract

According to the Home Literacy Model (Sénéchal and LeFevre, 2002, 2014), young children can be exposed to two distinct types of literacy activities at home. First, meaning-related literacy activities are those where print is present but is not the focus of the parent–child interaction, for example, when parents read storybooks to their children. In contrast, code-related literacy activities focus on the print, for example, activities such as when parents teach their children the names and sounds of letters or to read words. The present study was conducted to expand the Home Literacy Model by examining its relation with children’s engagement in literacy activities at home and at school as Finnish children transitioned from kindergarten to Grades 1 and 2. Two facets of children’s engagement were examined, namely, children’s independent reading at home and their interest in literacy activities. Children (*N* = 378) were tested and interviewed at the ends of kindergarten, Grade 1, and Grade 2. Mothers completed questionnaires on their home literacy activities at each test time, and they reported the frequency with which their children read independently twice when children were in grade school. Tested was a longitudinal model of the hypothesized relations among maternal home literacy activities (shared reading and teaching of reading), children’s reading skills, independent reading, and their interest in literacy activities/tasks as children progressed from kindergarten to Grade 2. Stringent path analyses that included all auto-regressors were conducted. Findings extended previous research in four ways. First, the frequency of shared reading and teaching of reading at home predicted the frequency of children’s independent reading 1 year later. Second, children with stronger early literacy skills in kindergarten read independently more frequently once they were in Grade 1. Third, parents adapted, from kindergarten to Grade 1, their teaching behaviors to their children’s progress in reading, whereas shared reading decreased over time. Fourth, children’s own reports of interest in literacy activities were mostly not linked to other variables. Taken together, these results add another layer to the Home Literacy Model.

## Introduction

Parents contribute to their children’s reading acquisition by exposing them to a rich home literacy environment (Torppa et al., 2006; Manolitsis et al., 2011; Niklas and Schneider, 2013). The types and the frequency of literacy activities at home prior to formal schooling have been linked longitudinally to the development of children’s reading acquisition by enhancing children’s language and early literacy skills (Sénéchal et al., 1998; Hood et al., 2008). Whereas most studies focused on home literacy activities before the start of formal schooling or Grade 1, fewer studies examined changes in home literacy activities once children enter formal schooling (e.g., Silinskas et al., 2012; Sénéchal and LeFevre, 2014). Moreover, recent concurrent and longitudinal evidence suggests that children’s engagement in literacy activities also plays a role in the relation between the home literacy environment and children’s reading skills (Sénéchal, 2006; Silinskas et al., 2012, 2013). The present study was conducted to expand the Home Literacy Model (Sénéchal and LeFevre, 2002) by examining its relation with children’s engagement in literacy activities at home and at school as Finnish children transitioned from kindergarten to Grades 1 and 2. Two facets of children’s engagement were examined, namely, children’s independent reading at home and their interest in literacy activities.

### The Home Literacy Model

According to the Home Literacy Model (Sénéchal et al., 1998; Sénéchal and LeFevre, 2002), young children can be exposed to two distinct types of literacy activities at home. First, meaning-related literacy activities (also often labeled as informal literacy activities) are those where print is present but is not the focus of the parent–child interaction, for example, when parents read storybooks to their children. In contrast, code-related literacy activities at home (often labeled as formal literacy activities) focus on the print, for example, activities such as when parents teach their children the names and sounds of letters or to read words. Meaning-related activities predict children’s reading acquisition indirectly by enhancing language development, whereas code-related activities predict reading indirectly by enhancing children’s early literacy skills. Support for this model has been found in opaque orthographies, such as English (Hood et al., 2008; Sénéchal and LeFevre, 2014) and French (Sénéchal, 2006) as well as some support in transparent orthographies, such as Greek (Manolitsis et al., 2011; Manolitsis et al., 2013), Lithuanian (Silinskas et al., submitted), German (Lehrl et al., 2013; Niklas and Schneider, 2013, 2017; Rose et al., 2018) and Finnish (Torppa et al., 2006, 2007; Silinskas et al., 2012; Silinskas et al., 2020). Although the Home Literacy Model postulates that parental literacy activities at home enhance children’s literacy outcomes, other evidence has shown that children’s own reading as well as children’s interest in literacy activities predict their literacy outcomes (Levin et al., 1997; Pomerantz and Eaton, 2001; Martini and Sénéchal, 2012; Silinskas et al., 2012, 2013; Torppa et al., 2019).

#### Children’s Independent Reading

Children’s independent reading can be defined as the frequency with which children voluntarily read on their own in anticipation of the satisfaction that is obtained from reading (Frijters et al., 2000; Leppänen et al., 2005; Clark and Rumbold, 2006). Although the term independent reading is used herein, other interchangeable terms include reading for pleasure (Sénéchal, 2006), voluntary reading (Krashen, 2004), leisure reading (Torppa et al., 2019), and a child’s own reading outside school/out-of-school reading habits (Silinskas et al., 2013).

Concurrent evidence suggests that children’s independent reading is positively related to their reading skills (for a review, see Schiefele et al., 2012). In a meta-analysis, Mol and Bus (2011) found seven studies, representing 517 Grades 1 and 2 children that included correlations between exposure to print through reading and word recognition. For these studies, print exposure was moderately, but significantly, associated with children skills (Mean *r* = 0.33). Yet, a limited number of short-term longitudinal studies showed a somewhat stronger association from skills to independent reading than the other way around (e.g., Aarnoutse and van Leeuwe, 1998). For instance, Leppänen et al. (2005) showed stronger cross-lagged paths from Grade 1 reading skills to Grade 2 frequency of independent reading than from independent reading to reading skills. In another study, word-reading skills in Grade 1 predicted independent reading of books in Grade 2, not the other way around (Torppa et al., 2019). In older children, Harlaar et al. (2011) also found a significant cross-lagged effect from Grade 5 reading skills (a composite of accuracy and comprehension) to the Grade 6 frequency of independent reading, but the reverse path was not significant.

Examination of the longitudinal relation between children’s independent reading and home literacy practices has been limited to a single study that showed that parental shared reading in kindergarten predicted children’s reports of independent reading for pleasure in Grade 4 after controlling for parent education, kindergarten early skills, Grade 1 reading, as well as Grade 4 reading comprehension (Sénéchal, 2006). In contrast, parent teaching in kindergarten did not predict the frequency of reading for pleasure. One goal of the present research was to investigate the reciprocal associations among parental home literacy activities and children’s independent reading. In contrast to Sénéchal who assessed independent reading only in Grade 4, in the present study independent reading was measured at the end of Grades 1 and 2. The rationale was that given the transparency of Finnish, children might become autonomous readers earlier (Lerkkanen et al., 2004) than in opaque languages such as in English or French. Moreover, this early autonomy might be predicted not only by shared reading, but also by parent teaching.

#### Children’s Interest in Literacy

Child interest can be defined as the perceived intrinsic value of a task, namely, the degree to which a task is enjoyable (Eccles et al., 1993). Certainly, children’s emotional engagement in reading activities is a key component of their interest to read (De Naeghel et al., 2012). In the present study, we examined how much children liked doing literacy activities at home and at school (Lerkkanen et al., 2012). Nurmi and Aunola (2005) reported that most young Finnish children in their study (*N* = 211) generally enjoyed doing literacy activities, with less than 16% of them reporting low interest in literacy tasks across the beginning and ends of Grades 1 and 2.

Studies provide mixed evidence on the links between children’s interest in literacy and their literacy outcomes. For example, in their meta-analysis of 26 correlational studies examining young children’s interest and literacy outcomes, Dunst et al. (2011) showed that children’s interest was positively associated with their alphabet knowledge (8 studies: Mean effect size = 0.14, 95% CIs: 0.08–0.20) as well as word recognition (7 studies: Mean effect size = 0.32, 95% CIs: 0.28–0.35). Also, 5-year-old children’s reports of interest in literacy activities contributed unique variance to alphabet knowledge after controlling for parent education, child gender and vocabulary (Baroody and Diamond, 2012). In contrast, other studies reported that finding links between child interest and child literacy skills was challenging. For instance, Kikas et al. (2015) did not find that child interest predicted child reading longitudinally in their cross-over analyses of data from 334 Estonian children. Also, Walgermo et al. (2018) did not find a direct link between children’s interest and their emergent literacy skills in a large sample of 1171 Norwegian children.

Empirical evidence on the relations between child interest in literacy and their home literacy environment is scarce. Based on a few reports that are available, child’s interest has been found to be positively related to their home literacy environment (Martini and Sénéchal, 2012; Hume et al., 2016; Carroll et al., 2019). For instance, concurrent and longitudinal associations were found between exposure to book reading (e.g., the amount of children’s books, shared reading, and children observing parent read) and children’s interest in books, whereas parent teaching literacy (e.g., teaching letters, pointing out words, and playing rhyming games) was concurrently and longitudinally associated with children’s interest in the alphabet and words (Hume et al., 2015).

Finally, some researchers reported positive associations between children’s interest and their reading independently from Grade 1 to Grade 2 (Frijters et al., 2000; Dunst et al., 2011; Baroody and Diamond, 2012). Consequently, we investigated this interrelation. In addition, we explored associations between children’s interest in literacy, their literacy outcomes, and their parents’ home literacy activities. However, due to mixed and scarce previous findings and due to the tendency of the young children to report liking literacy activities, we did not set any specific hypotheses.

#### Longitudinal Changes in Home Literacy Activities

Studies examining changes in home literacy practices as children transition into school revealed novel patterns of associations. In a sample of English-speaking children schooled in French, Sénéchal and LeFevre (2014) found that the frequency of parent teaching and expectations about literacy in kindergarten positively predicted growth in English literacy skills from kindergarten to the beginning of Grade 1. Moreover, parent teaching and listening to their children read at the beginning of Grade 1 positively predicted child reading skills in English at the end of Grade 1 after controlling for beginning of Grade 1 reading, phoneme awareness, and vocabulary. In sharp contrast, parent teaching and listening to the child read in Grade 1 was negatively related to reading skills at the end of Grade 2. Importantly, child reading skills at the beginning of Grade 1 was a negative predictor of parent teaching/listening at the end of Grade 2. Sénéchal and LeFevre interpreted these findings as an indication that parents were responsive to their children’s reading skills in that they provided more support when their children had more difficulty reading. In fact, parents who increased their teaching from Grade 1 to 2 had children with lower reading scores at the end of Grade 1 as compared to parents who maintained or who decreased their teaching.

The longitudinal associations between children’s reading skills and parental involvement may differ as a function of orthographic transparency. For instance, English is an opaque orthography because it has multiple exceptions to phoneme-grapheme connections (e.g., the phonology of ea in bear vs. beard) whereas Finnish is a transparent orthography in which letters consistently map on to the sound of the spoken language. It is well established that differences in transparency across languages affect the speed of children’s reading acquisition (Ziegler et al., 2010). Due to the transparency of the Finnish orthography paired with phonics instruction, children learn to read very fast in comparison to children in many other countries (Seymour et al., 2003; Silinskas et al., 2010b; Silinskas, 2012). In fact, the vast majority of Finnish children master decoding during the first half of Grade 1 (Lerkkanen et al., 2004). Because learning to read in Finnish is easier than learning in English, one could anticipate that parents might become responsive to their children’s reading behaviors earlier. There is some evidence that this is the case (Silinskas, 2012; Silinskas et al., 2015). In Finnish samples, the parent–child home literacy activities were positively correlated with children’s skills in kindergarten, whereas the relation became negative in Grade 1 (Silinskas et al., 2010a, 2012). This evidence supports the notion that one needs to consider orthographic transparency when studying the reciprocity between skills and home literacy activities.

#### The Present Study

In Finland, compulsory education (Grade 1) begins in the year of the child’s seventh birthday. Immediately before Grade 1, children attend kindergarten for 1 year. The main objectives of the kindergarten curriculum emphasize children’s personal and social growth. Although emerging literacy skills are not systematically taught, they are promoted by playful activities involving letters, phonological awareness, and shared reading activities. Once children enter Grade 1, they receive 7 h of literacy teaching per week focusing on learning to decode and practice fluency and comprehension. Because decoding reaches a high level of accuracy for most Grade 1 students after only a few months of school (Lerkkanen et al., 2004), students’ commitment and motivation for silent reading is supported daily from Grade 1 onward. Gains in reading fluency and comprehension are encouraged by the availability of high-interest texts at multiple levels of difficulty and by giving students the freedom to choose reading materials. Children are also given time to read what they choose, without being evaluated (Torppa et al., 2016).

The goal of the present study was to investigate, in a large sample of Finnish families, the longitudinal interplay among mothers’ reports of home literacy activities (shared reading and teaching of reading), children’s independent reading, their interest in reading, and their reading skills during the transition to primary school. Mothers completed questionnaires about home literacy activities, and children’s skills and interest were assessed at the ends of kindergarten, Grade 1, and Grade 2. Prior to testing the predicted longitudinal links, it was necessary to verify whether the relations among the kindergarten variables were consistent with the two key components of the Home Literacy Model. First, mothers’ reported frequency of teaching to read in kindergarten should be positively linked to children’s early literacy, but not to vocabulary. In contrast, mothers’ reported frequency of shared reading in kindergarten should be positively linked to children’s vocabulary, but not to early literacy (Sénéchal et al., 1998).

**FIGURE 1:**
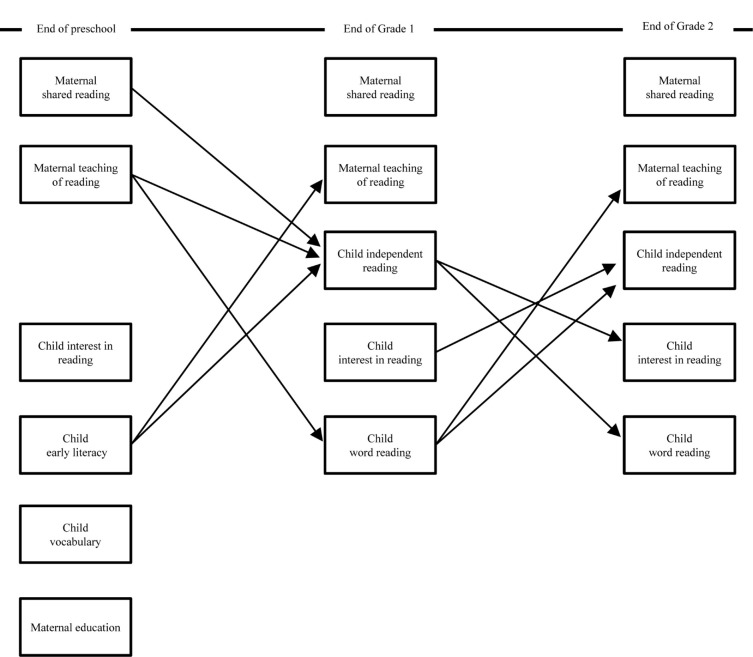
Hypothesized model relating maternal home literacy activities and child word reading, independent reading, and interest after controlling for child vocabulary and maternal education (*N* = 378). Arrows depicting correlations and auto-regressors were omitted for simplicity.

Listed below are six longitudinal predictions, based on past research, that led to the hypothesized theoretical model presented in Figure 1. This model included all auto-regressors of parent and child measures. Of special note, when longitudinal predictions were based on findings obtained in English, the timeline was shortened to reflect the documented rapid reading gains made by Grade 1 Finnish children.

(1) Shared reading and parent teaching in kindergarten should be positively linked to Grade 1 children’s independent reading at home (Leppänen et al., 2005; Sénéchal, 2006; Hume et al., 2015). This link should only be present from kindergarten to Grade 1 given the rapidity with which Finnish children acquire reading skills as well as the inclusion of all available auto-regressors in the model.
(2) Parent teaching in kindergarten should be linked to growth in reading skills in Grade 1 and in turn Grade 1 reading skills should be linked negatively to the frequency of parent teaching in Grade 2 (Silinskas et al., 2010a, 2013).
(3) The strength of children’s early literacy skills should influence the frequency of parent teaching at the end of Grade 1 (Silinskas et al., 2012; Sénéchal and LeFevre, 2014). Specifically, children’s early literacy skills feed back onto parent teaching such that the relation becomes negative: Parents of children with greater early literacy skills in kindergarten age should report teaching less at the end of Grade 1, and vice versa.
(4) Children’s early literacy skills in kindergarten should be positively linked to the frequency with which they read independently at home after 1 year of formal schooling (i.e., end of Grade 1; Frijters et al., 2000; Baroody and Diamond, 2012).
(5) There should be positive cross-over links between reading skills and reading independently from Grade 1 to Grade 2 (Leppänen et al., 2005; Torppa et al., 2019).
(6) In addition, there should be positive cross-over links between children’s interest and their reading independently from Grade 1 to Grade 2 (Frijters et al., 2000; Dunst et al., 2011; Baroody and Diamond, 2012; Martini and Sénéchal, 2012).

When testing the hypothesized model, child vocabulary and maternal education were included because these two variables were associated with reading skills and home literacy activities in previous studies (e.g., Frijters et al., 2000; Torppa et al., 2006, 2007; Baroody and Diamond, 2012; Carroll et al., 2019).

An ancillary goal of the present research was to understand better the predicted negative association between mother teaching and child reading. To do so, we tested the hypothesis that Finnish mothers who were responsive to their children’s reading skills increased their teaching when their children had lower reading skills, whereas mothers of children with higher literacy skills either decreased or maintained the same teaching frequency (Sénéchal and LeFevre, 2014).

## Methods

### Participants and Procedure

The data came from a large-scale longitudinal study of approximately 2,000 children followed from kindergarten to Grade 9, their parents and teachers (Lerkkanen et al., 2006–2016). The subsample selected for the present study included all the 378 children (179 girls, 199 boys) for whom children’s interest in reading was assessed and for whom mothers were asked to complete questionnaires about their children’s independent reading. The participants were recruited from four Finnish municipalities. Only children with written parental consent were tested. As is typical of the school population in Finland, the sample was highly homogeneous in ethnic and cultural background. All children lived in families where Finnish language was spoken at home, and only 3% of children also spoke an additional language at home (e.g., English, Russian, and Swedish).

#### Children

The children (*M*age = 67.7 months, *SD* = 3.4, at the first measurement point) were followed across three time-points: at the ends of kindergarten (April; *N* = 377; K), Grade 1 (April; *N* = 377; G1), and Grade 2 (April; *N* = 365; G2). In kindergarten (K), children were individually tested on their emergent literacy and vocabulary skills and children were individually interviewed on their interest in reading activities. In Grades 1 and 2, one reading test (ALLU) was administered in group/classroom situations, whereas the other reading test (Lukilasse) and the interview concerning reading interest were conducted individually. Although the sample size was slightly different at each measurement point (e.g., due to children missing a testing session), no systematic differences were observed between participants who had participated in the study and those whose data was not available at certain time-point.

#### Mothers

Mothers (ages ranged from 24 to 55; *M* = 37.4, *SD* = 5.2) answered questionnaires at three time points: the ends of kindergarten (K, April; *N* = 338), Grade 1 (G1; April; *N* = 283), and Grade 2 (G2, April; *N* = 289). 88% of mothers reported on their educational attainment and the distribution was as follows: 2.8% of mothers had no vocational education after the 9-year compulsory schooling; 1.4% completed a short vocational course; 25.4% had a vocational school qualification; 23.9% had a vocational college qualification; 14.1% a polytechnic degree or a Bachelor’s degree; 27.3% a Master’s degree; and in 5.1% of mothers had a licentiate or doctoral degree. This distribution is representative of the attainment in Finland (Statistics Finland, 2007). Mothers also reported on family composition: 77.1% of the children lived in families with two parents; 9.8% of the children lived in families where the mother or father had a new spouse and children; 11.6% of the children lived with a single mother; and 1.5% of the children lived in families with shared parenthood after divorce or separation. The number of children in a family ranged from one to nine (*M* = 2.42, *SD* = 1.20); 22.3% of the mothers reported being unemployed.

## Materials

The measures’ psychometric properties, including scale reliabilities, were calculated for the sample (*N* = 378) and presented in Table 1.

**TABLE 1:** Descriptive statistics and psychometric properties at the ends of kindergarten (K), Grade 1 (G1), and Grade 2 (G2).

Variable	*n*	*M*	*SD*	*ICC*	Reliability Cronbach α	Range		Skewness
						Potential	Actual	
**Home learning environment**								
Shared reading (K)	336	2.88	1.18	0.012		1–5	1–5	–0.12
Shared reading (G1)	282	2.63	1.10	0.064		1–5	1–5	0.05
Shared reading (G2)	289	2.21	1.02	0.055		1–5	1–4	0.56
Teach reading (K)	338	2.20	0.99	0.099		1–5	1–5	0.49
Teach reading (G1)	279	2.32	1.21	0.154		1–5	1–5	0.64
Teach reading (G2)	289	1.59	1.03	0.067		1–5	1–5	1.88
**Child measures**								
Vocabulary (K)	377	20.07	3.28	0.081	0.61	1–30	8–29	–0.35
Early literacy								
Alphabet (K)	377	23.29	6.38	0.020	0.95	1–29	1–29	–1.38
Word reading (K)	376	3.81	4.27	0.112	0.92	1–30	0–10	0.55
Word reading^a^								
Test 1 (G1)	377	18.50	9.02	0.082	0.97	1–80	0–50	0.63
Test 1 (G2)	369	28.20	12.04	0.032	0.97	1–80	0–66	0.00
Test 2 (G1)	365	24.78	7.49	0.036	0.98	1–90	3–58	0.39
Test 2 (G2)	358	40.63	9.67	0.041	0.98	1–90	2–75	–0.21
Interest in reading^b^ (K)	377	3.93	0.99	0.122	0.63	1–5	1–5	–0.97
Interest in reading^b^ (G1)	369	3.81	1.01	0.059	0.80	1–5	1–5	–0.77
Interest in reading^b^ (G2)	358	3.75	0.91	0.045	0.78	1–5	1–5	–0.69
Independent reading^c^ (G1)	282	2.47	0.85	0.058	0.73	1–5	1–5	0.63
Independent reading^c^ (G2)	288	2.67	0.86	0.046	0.72	1–5	1–5	0.33

**^*a*^Test 1 = the group-administered word-reading subtest of the ALLU test battery; Test 2 = the individually administered Lukilasse test. ^*b*^Averaged scores on the three questions asked of children on their liking literacy activities in general, at home, and at school. ^*c*^Mothers reported frequency of their children’s independent reading at home averaged across four book types*.*

### Maternal Questionnaire

#### Home Literacy Activities (K, G1, and G2)

The questionnaire on home literacy activities, from this large-scale study (Lerkkanen et al., 2006–2016), was based on the work of; Sénéchal et al. (1998), Sénéchal and LeFevre (2002), and Sénéchal (2006). The questions captured both current and retrospective frequency of home literacy activities.

*Shared reading* was assessed by asking mothers to report on the frequency with which they read to or with their child. In kindergarten, shared reading was assessed by *How often do you read books to your child or together with your child?* A five-point scale was used (1 = *less than once a week*; 2 = 1–3 *times a week*; 3 = 4–6 *times a week*; 4 = *once a day*; and 5 = *more than once a day*). In Grades 1 and 2, the question was *How often does the mother read a book or a newspaper/magazine with the child?* Mothers responded on a five-point scale [1 = *not at all or rarely;* 2 = *once or twice a week* (*on 1 to 2 days*)*;* 3 = *several days a week* (*on 3 to 6 days*)*;* 4 = *once a day/daily;* 5 = *several times a day*]. Although measuring the frequency of shared reading by a single item does not capture the richness of informal literacy activities at home, a meta-analysis showed that shared reading had similar relations to child language and literacy outcomes regardless of whether a single item or a composite of items was used (Bus et al., 1995).

*Teaching of reading* was assessed by asking mothers to report on the frequency they taught their child to read. In kindergarten, the question, *How often do you teach/have you previously taught your child to read*, was answered on a five-point scale with two defined anchors: 1 = *not at all/very rarely* to 5 = *very often/daily*. In Grades 1 and 2, the question was *How often do you teach your child to read?* that was answered on a five-point scale in Grade 1 (1 = *not at all;* 2 = *rarely;* 3 = *once or twice a week;* 4 = *several days a week;* 5 = *once a day/daily*) and a six-point scale in Grade 2 (0 = *not anymore; because the child has acquired the skill;* 1 = *never;* 2 = *rarely;* 3 = *once or twice a week;* 4 = *several days a week;* 5 = *every day*). To make the scale in Grade 2 similar to the scales in the previous time-points, the first two categories, 0 and 1, were combined. This decision was based on the similarity of the meaning between these two categories, namely, that parents are not teaching. Also 4.2% of mothers answered “1 = not at all” in Grade 1, and the combination of the two points in Grade 2 resulted in a similar percentage. Although measuring constructs with a single item is not optimal, teaching of reading is the key aspect of code-related home literacy activities for the children of this developmental stage. For instance, Martini and Sénéchal (2012) showed that it was the teaching of this higher level skill that was associated with child literacy outcomes. Moreover, Aunola and Nurmi (2007) reported that daily parental reports of reading-related teaching and the overall parental perception of the frequency of their teaching of reading in Grade 1 correlated by 0.28 (*p* < 0.01).

#### Children’s Independent Reading (G1 and G2)

The frequency of independent reading can be reported by children (Sénéchal, 2006), parents (Silinskas et al., 2013), and teachers and observers (Baroody and Diamond, 2013). We relied on parental reports due to the young age of the children and because we were interested in the frequency of the reading instances outside school. When their children were in Grades 1 and 2, mothers answered four questions on children’s independent reading: *How often does your child do the following things: My child independently reads* (1) *comics or children’s magazines*, (2) *picture books*, (3) *unillustrated books, and* (4) *non-fiction books* (*for instance, about animals*). Answers were provided on a five-point scale: 1 = *not at all or rarely*; 2 = *once or twice a week* (*1–2 days*); 3 = *several days a week* (*3–6 days*); 4 = *once a day/daily*; and 5 = *several times a day*. The average of the four items was used in the analyses.

### Child Measures

#### Interest in Reading (K, G1, and G2)

At each time point, children’s interest in reading was assessed with the Task Value Scale for Children (TVS-C; Nurmi and Aunola, 1999; Nurmi and Aunola, 2005). The scale is based on the ideas of Eccles et al., 1993, and has been also used in studies among Finnish 6- to 7-year-old children (Lerkkanen et al., 2012; Viljaranta et al., 2014). This scale consists of three items measuring children’s interest in activities and tasks involving letters in kindergarten and reading in Grades 1 and 2: (1) *How much do you like letter/reading activities?*; (2) *How much do you like doing letter/reading tasks in kindergarten/in school?*; (3) *How much do you like doing letter/reading tasks at home?* During testing, the questions were read aloud to the children, and children were presented with a set of five faces drawn to depict an assessment scale ranging from a big frown (i.e., very negative) to a big smile (i.e., very positive). After each question, the children were asked to point to the picture that best described the liking of a particular reading task (1 = *“I do not like it at all/I dislike doing those tasks”*; 5 = *“I like it very much/I really enjoy doing those tasks”*). An average score of the three items was used in the subsequent analyses. Prior to testing, the task was explained and children practiced indicating their interest in three practice items (e.g., sports and music) to ensure that children understood the procedure/task.

#### Early Literacy (K)

Early literacy was assessed individually with two subtests from the ARMI test battery (Lerkkanen et al., 2006–2016). First, a letter-naming test required children to name all 29 uppercase letters of the Finnish alphabet, presented in a random order. Second, a test of reading accuracy was administered where children were asked to read 10 uppercase words. The words were of increasing difficulty; children were given as much time as needed to read the words accurately. The word reading test was discontinued after three unsuccessful attempts.

#### Reading Skills (G1 and G2)

Reading was assessed with two tests. First, children were assessed with the group-administered reading-fluency subtest of a nationally normed reading test battery (ALLU; Lindeman, 1998). The subtest included 80 items, each of which consisted of a picture with four phonologically similar words attached to it. The child read the four words silently, after which he or she had to draw a line between the picture with and the word semantically matching it. The final score was the number of answers completed correctly in a 2-min time limit. Second, an individually administered word-list reading test was used (Lukilasse test for 6- to 12-year-old children; Häyrinen et al., 1999). A child was presented with a list of 90 real words divided into four columns. The words ranged from 1- to 7-syllabic word forms, written in lowercase letters. The child was instructed to read the words aloud; the final score was the number of words read correctly within a 45-s time limit.

#### Vocabulary

In kindergarten, children’s receptive vocabulary was assessed with a 30-item shortened version of the Peabody Picture Vocabulary Test-Revised (PPVT-R, Form L; Dunn and Dunn, 1981). The tester said a word, and children had to select which one of four pictures correctly represented the spoken word. The items of the shortened version were selected to represent a range of difficulty levels based on the data from the full-scale administration of the PPVT-R in the Jyväskylä Longitudinal Study of Dyslexia (see Lyytinen et al., 2004). Each correct response received one point (max. 30).

### Data Analysis Strategy

A path model was used to test the hypothesized model, with all the analyses run with the Mplus statistical package, version 8 (Muthén and Muthén, 1998–2010). Missing data for the study variables ranged from 0.3 to 26.2% (*M* = 11.1%, *SD* = 10.5%), and these data were not missing completely at random based on Little’s (1988) MCAR test (χ^2^ [424] = 481.08, *p* = 0.03). Attrition analyses between kindergarten (K) and Grade 1 (G1) revealed that mothers of children with better vocabulary skills tended to stay in the study (Δ*M* = −1.28, *p* < 0.01, Cohen’s *d* = 0.39), whereas there were no systematic differences on any of the study’s variables between Grade 1 (G1) and Grade 2 (G2). Given that vocabulary was a control variable rather than an outcome and given the lack of systematic differences on parent reports and child literacy and interest, we applied the standard full-information maximum likelihood (FIML) method to account for missingness. This method takes all available data to estimate the model without imputing data. Some variable distributions were skewed. Therefore, the model parameters were estimated using the MLR estimator (maximum likelihood with robust standard errors). This estimator is implemented in Mplus and produces chi-square test statistics and standard errors for missing data with non-independent observations and non-normally distributed variables.

The data were nested because children came from 151 classes. Therefore, intra-class correlations (ICCs) were calculated to estimate the effect of classroom membership. The ICC represents the proportion of the total score variance that is attributable to an individual’s membership in a particular class. As presented in Table 1, the ICCs across all measures varied from 0.012 to 0.154 (from *p* > 0.05 to *p* < 0.001). Because some ICCs were statistically significant, the Mplus TYPE = COMPLEX option was used to include kindergarten classrooms as a clustering variable. This resulted in the computation of corrected standard errors and the calculation of model fit tests that took the nested structure of the data into account.

Following Hu and Bentler (1999), model fit was examined with a combination of indices in order to minimize Type I and Type II errors. Three fit indices, appropriate for large samples, were used and evaluated based on the criteria suggested by Hu and Bentler to be indicative of a good model: Comparative Fit Index (CFI) > 0.95, root mean square error of approximation (RMSEA) < 0.06, and standardized root mean square residual (SRMR) < 0.08.

## Results

The descriptive data, including scale reliabilities, are presented in Table 1. On average, Finnish mothers indicated reading books to their children up to six times a week in kindergarten and Grade 1, and then reduced their reading to once or twice a week in Grade 2. As for teaching their children to read, mothers reported, on average, that they taught their children but did so infrequently. Children indicated, at each test point, that they liked doing letter tasks or reading in school, selecting, on average, the smiley face on each of the three questions at each test point. Finally, mothers reported, on average, that their children read on their own several days a week in Grades 1 and 2.

Examination of the zero-order correlations in Table 2 are in accord with the Home Literacy Model (Sénéchal and LeFevre, 2002). First, the mother-reported frequency of shared reading in kindergarten was positively related to children’s vocabulary, but not to early literacy. Of note, the correlation between shared reading and vocabulary remained significant after controlling for parent education, partial *r* = 0.20, *p* < 0.01. Second, the mother-reported frequency of teaching in kindergarten was positively and longitudinally related to literacy outcomes across time, whereas shared reading was not related to literacy outcomes or was related negatively. Third, the expected change from positive to negative associations between teaching and literacy outcomes was found once children were in grade school. Examining the stability of home literacy activities across time is also informative. Interestingly, shared reading behaviors seemed more stable over time with correlations between 0.58 and 0.69 across kindergarten to Grade 2. In contrast, reports of teaching in kindergarten were not correlated with subsequent teaching (*r*s < 0.06), whereas the correlation increased to 0.46 between Grades 1 and 2. This latter pattern suggests changes in teaching behaviors across families during the transition to grade school.

**TABLE 2:** Concurrent and longitudinal correlations^a^ among home literacy variables and child measures at the ends of kindergarten (K), Grade 1 (G1), and Grade 2 (G2).

	Shared Reading			Teach Reading			Voc.	Early Lit.	Word Reading		Reading Interest			Ind. Reading	
	K	G1	G2	K	G1	G2	K	K	G1	G2	K	G1	G2	K	G1
**Home learning environment**															
Shared reading (K)	−														
Shared reading (G1)	**0.69**	−													
Shared reading (G2)	**0.58**	**0.66**	−												
Teaching of reading (K)	**0.14**	0.10	0.04	−											
Teaching of reading (G1)	0.08	**0.28**	**0.18**	0.05	−										
Teaching of reading (G2)	0.03	0.10	**0.22**	0.02	**0.46**										
**Children’s measures**															
Vocabulary (K)^a^	**0.22**	**0.16**	**0.15**	–0.02	**−0.15**	**−0.13**	−								
Early literacy (K)	0.10	–0.08	–0.05	**0.31**	**−0.46**	**−0.34**	**0.29**	−							
Word reading (G1)	–0.06	**−0.21**	**−0.26**	**0.16**	**−0.47**	**−0.32**	**0.17**	**0.62**	−						
Word reading (G2)	0.03	–0.10	**−0.19**	0.08	**−0.44**	**−0.35**	**0.22**	**0.55**	**0.81**	−					
Reading Interest (K)	0.06	–0.02	0.06	–0.01	–0.04	0.05	–0.01	0.05	0.02	0.02	−				
Reading Interest (G1)	–0.03	0.01	–0.07	–0.05	–0.03	0.01	0.01	0.03	0.09	0.08	**0.34**	−			
Reading Interest (G2)	0.06	–0.02	0.04	0.09	0.03	0.02	–0.05	0.08	0.05	0.02	**0.23**	**0.38**	−		
Independent reading (G1)	**0.15**	0.05	–0.01	**0.22**	**−0.18**	**−0.16**	0.09	**0.32**	**0.39**	**0.33**	–0.01	0.06	**0.18**	−	
Independent reading (G2)	**0.21**	0.07	0.01	**0.21**	–0.12	–0.10	0.10	**0.20**	**0.24**	**0.28**	0.10	0.04	**0.13**	**0.65**	*–*
**Control variable**															
Maternal education	**0.17**	0.11	0.13	0.02	–0.12	–0.05	**0.20**	**0.16**	**0.12**	0.10	–0.02	0.04	**0.13**	0.11	0.03

**Voc., vocabulary; Lit., literacy; Ind., independent. Correlations in bold are statistically significant (r = 12.4, p < 0.05; r = 0.16, p < 0.01). ^*a*^The correlation between shared reading and vocabulary remained significant after controlling for parent education, partial r = 0.20, p < 0.01.**

Novel findings concerned children’s autonomous reading at home. Here, both shared reading and teaching in kindergarten were positively and longitudinally associated with children’s independent reading. Moreover, children’s independent reading was positively, longitudinally, and reciprocally associated with literacy skills.

Additional novel findings concerned children’s own reports of how much they liked doing literacy activities in kindergarten and school. Unexpectedly, child interest in literacy activities at school was nearly not associated with any of the other variables tested. In fact, only two small coefficients were significant of the 36 correlations between interest and other measures. Given that the probability of obtaining spurious results was 1.8 tests out of 36 conducted, then these two significant coefficients could be due to chance. Therefore, it was decided not to analyze interest further, and consequently, interest measures were removed from the path model.

### Testing the Home Literacy Model

**FIGURE 2:**
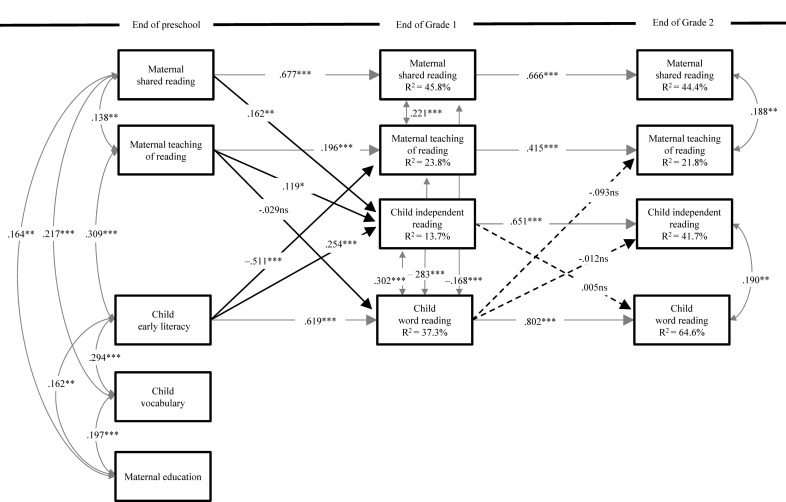
Standardized parameter estimates of the hypothesized model. Gray paths indicate correlations and auto-regressors; black solid paths are statistically significant; and black dashed paths are not. ^∗^*p* < 0.05, ^∗∗^*p* < 0.01, ^∗∗∗^*p* < 0.001.

Figure 2 depicts the standardized parameter estimates for the longitudinal links from kindergarten to Grade 2 for the hypothesized model once child interest measures were excluded. Importantly, the indices of the model met the Hu and Bentler (1999) criteria indicating good model fit, CFI = 0.96, SRMR = 0.05, RMSEA = 0.06, 95% CIs [0.05–0.08].

The predicted cross-over links between teaching and literacy skills from kindergarten to Grade 1 were partially supported. Although parental teaching of reading during kindergarten was not predictive of word reading 1 year later, mothers were responsive to their children’s early literacy skills as evidenced by the negative and moderately strong path from early skills in kindergarten to the frequency of mother teaching at the end of Grade 1 over and above the auto-regressor. That is, mothers of children who had weaker early literacy skills seem to have increased their teaching. This level of responsiveness was not found between Grades 1 and 2 because reading skills at the end of Grade 1 did not predict change in the frequency of mother teaching 1 year later.

As expected, mothers’ reported frequency of shared reading and teaching reading in kindergarten positively predicted child independent reading at the end of Grade 1. The more mothers reported engaging in shared reading and teaching reading in kindergarten, the more frequently they reported, 1 year later, that their children were involved in independent reading activities at home. Moreover, stronger early literacy in kindergarten also predicted children’s independent reading at the end of Grade 1. However, the predicted cross-over links between the frequency independent reading and word reading across Grades 1 and 2 were not statistically significant.

Multicollinearity is a common problem in correlational research, thus we investigated its manifestation in our data. Overall, the non-autoregressive correlations (correlations between different constructs) were moderate at best (highest correlation between word reading in Grade 1 and teaching of reading in Grade 1; *r* = –0.47, p < 0.01). We also calculated tolerance levels and the variance inflation factor (VIF) for the variables in Figure 2. The obtained tolerance levels ranged from 0.354 to 0.542 and the VIF ranged from 1.845 to 2.824. These ranges are below those suggested to be indicative of multicollinearity (see O’Brien, 2007). Therefore, multicollinearity was not an issue.

Finally, to make sure that including/excluding interest would not have an influence on our reported results (Figure 2), we ran analyses where interest was included to the model. In particular, stabilities of interest across time were specified, as were the significant correlations from Table 2 and all concurrent associations between all variables within each measurement point. Including interest to the model did not change the results reported in Figure 2 (i.e., results were the same), thus providing one more justification to exclude interest from our final model.

### Mothers’ Responsiveness to Their Children’s Reading Skills

As did Sénéchal and LeFevre (2014), we assessed whether the patterns of negative associations between maternal home literacy activities and children’s reading skills were such that mothers of children with lower literacy skills increased their home literacy activities over time whereas mothers of children with higher literacy skills either decreased or maintained home literacy activities. The analyses included participants with no missing values on maternal reports for teaching of reading and shared reading (*N* = 253). Then, we divided the sample in four equal groups (i.e., 25% of participants in each group) based on children’s word-reading skills at the end of Grade 2 (G2). The groups were therefore labeled as poor, below average, above average, and good readers. Doing so allowed us to investigate change in maternal teaching of reading with a mixed-design ANOVA. Time, as a within-subject variable, included three levels: the ends of kindergarten (K), Grades 1 (G1), and Grade 2 (G2). Reading skills was the between-subject variable with four levels. The same design was used to explore change in shared reading.

**FIGURE 3:**
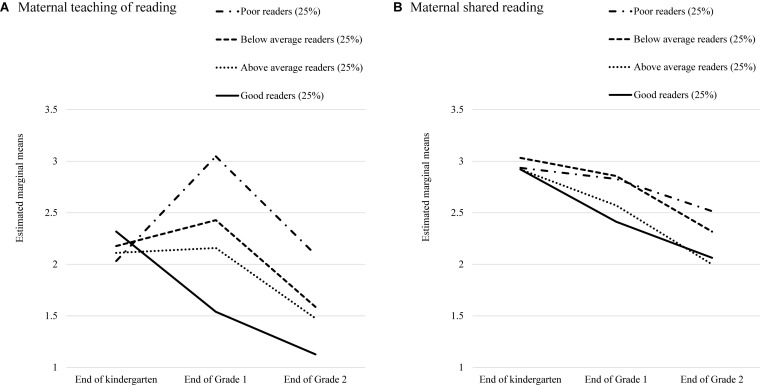
Estimated marginal means of the frequency of maternal teaching reading **(A)** and shared reading **(B)** at three time points as a function of children’s word-reading skills at the end of Grade 2.

The analysis for teaching revealed significant main effects of Time (*F*[2,492] = 44.97, *p* < 0.001; $\eta_{\text{p}}^{2}$ = 0.16) and Reading Skills (*F*[3,246] = 12.22, *p* < 0.001; $\eta_{\text{p}}^{2}$ = 0.13). However, these two main effects have to be interpreted in light of a significant interaction between Time and Reading Skills (*F*[6,492] = 10.41, *p* < 0.001; $\eta_{\text{p}}^{2}$ = 0.11). *Post hoc* Bonferroni contrasts revealed differences across groups that help explain the change from positive to negative associations between children’s reading skills and maternal teaching. As shown in Figure 3A, mothers initially reported similar frequencies of teaching, but the patterns changed in grade school. Specifically, for poor readers the frequency of maternal teaching increased from kindergarten to Grade 1 (Δ*M* = –1.00, *S.E.* = 0.21, *p* < 0.001), whereas mothers of the good readers decreased the frequency of teaching at the end of Grade 1 (Δ*M* = 0.78, *S.E.* = 0.14, *p* < 0.001). In contrast, the frequency of teaching did not change significantly for below average readers (Δ*M* = –0.27, *S.E.* = 0.19, *p* = 0.47) and above average readers (Δ*M* = –0.05, *S.E.* = 0.17, *p* = 1.00). Although mothers of all four groups reported teaching less in Grade 2 than in Grade 1 (*p* < 0.001), mothers of poor readers and below average readers still reported teaching more than did mothers of good readers (Δ*M* = 0.97, *S.E.* = 0.17, *p* < 0.001 and Δ*M* = 0.46, *S.E.* = 0.17, *p* < 0.05, respectively).

As for shared reading, there was a significant effect of Time only (*F*[2,498] = 77.77, *p* < 0.001; $\eta_{\text{p}}^{2}$ = 0.24). *Post hoc* Bonferroni contrasts confirmed the pattern shown in Figure 3B: Mothers reported reading less in Grade 1 than in kindergarten (Δ*M* = 0.30, *S.E.* = 0.06, *p* < 0.001), and less at the end of Grade 2 than in Grade 1 (Δ*M* = 0.43, *S.E.* = 0.06, *p* < 0.001). The Reading Skills main effect was not significant (*F*[3,249] = 1.71, *p* = 0.17; $\eta_{\text{p}}^{2}$ = 0.02) nor did it interact with shared reading (*F*[6,498] = 1.97, *p* = 0.07; $\eta_{\text{p}}^{2}$ = 0.02). Here, we found that mothers of poorer readers reported reading more than mothers of better readers at the end of Grade 1 (Δ*M* = 0.35, *S.E.* = 0.14, *p* < 0.05), presumably because the stronger readers were reading independently.

## Discussion

The present study aimed at expanding the Home Literacy Model (Sénéchal and LeFevre, 2002, 2014) by examining the role of two child variables, namely, children’s interest in literacy activities at kindergarten/school/home and their independent reading at home. Finnish families and children were followed from kindergarten to Grade 2. The obtained findings demonstrated that parental practices and children’s literacy skills predict children’s independent reading rather than the reverse. There are four key findings. A first novel finding is that both maternal shared reading and teaching of reading in kindergarten positively predicted child independent reading at the end of Grade 1. Second, children with stronger early literacy skills in kindergarten read independently more frequently once they were in Grade 1. Third, the results extended to Finnish families previous findings showing that parents, in the early school years, adapt the frequency of their teaching of reading and shared reading to their children growing literacy skills. Fourth, children’s interest was not linked to other variables.

### Home Literacy Activities and Children’s Independent Reading

We found that literacy activities at home prior to school positively predicted the frequency with which Finnish children read independently at the end of Grade 1. The novelty of the finding stems from the fact that both shared reading as well as parent teaching were significant predictors. Previous findings with children acquiring reading in French (Sénéchal, 2006) had shown that shared reading, but not parent teaching, in kindergarten predicted the frequency of children reading on their own in Grade 4. The findings in the present study and in Sénéchal were robust because the models controlled for children’s literacy skills, child vocabulary, and parent education.

As children’s first educators, parents can stimulate their children’s interest in reading in different ways. Sénéchal (2006) suggested that shared reading is an enjoyable activity from which children may learn that reading is fun. Of course, parents who value shared reading may also value reading as a desirable activity, read for pleasure themselves, and may have a home library (for a review, see Sénéchal, 2012). Parents who teach early literacy skills can also facilitate children’s entry into the autonomous reading. In the present study, the small but significant concurrent correlation between these two types of literacy activities at home suggests that there is variability across Finnish families in what they choose to promote. In addition, the magnitude of the coefficients in the path model suggests that the impact of these two types of home activities over and above early literacy skills is modest but it has a lasting effect over and above the instruction that children received in Grade 1. This novel addition to the Home Literacy Model now requires replication in other orthographies.

### Children’s Independent Reading and Reading Skills

The frequency with which children read independently at the end of Grades 1 and 2 was positively correlated with children’s literacy skills at all time points. Yet, in our complex model, the only significant longitudinal path was from early literacy skills at the end of kindergarten to independent reading at the end of Grade 1. These results suggest that children who enter Grade 1 with stronger skills might become autonomous readers more quickly, or at the very least read more frequently, than children with weaker skills. Cunningham and Stanovich (1998) have provided sound evidence of this in their review. That the longitudinal effect was present only during the transition from kindergarten to Grade 1 might be due to the ease of learning to read in Finnish. For instance, previous reports showed that one third of Finnish children can read already at the end of kindergarten (Silinskas et al., 2010b), and most will read accurately by the end of the school year (Lerkkanen et al., 2004). Given this rapid progress, it might not be surprising that we did not find evidence of a relation between independent reading and reading skills across Grades 1 and 2 despite positive longitudinal correlations. In fact, Torppa et al. (2019) showed that the reciprocal relation between independent reading and word reading between Grades 1 and 2 was limited to reading comprehension, not word reading, when using the entire sample of about 2,000 children in the large study. It might be the case that the transparency of Finnish is such that it is higher level skills that show a robust association with reading frequency as children progress in grade school. Future research in transparent orthographies could examine whether this pattern also holds for higher-level measures, such as vocabulary and background knowledge, found to be associated with the frequency of autonomous reading in English (e.g., Hirsch, 2003; Mol and Bus, 2011).

### Mothers’ Responsiveness to Their Children’s Reading Skills

In contrast to Sénéchal and LeFevre (2014), we did not find that the frequency of mother reports of teaching at the end of kindergarten was linked longitudinally to growth in children’s reading skills from the end of kindergarten to the end of Grade 1. In Sénéchal and LeFevre, however, a significant path between teaching and growth in early literacy was found when measuring teaching and literacy at the beginning of the school years. As such, the English-speaking children in their study had not received much formal instruction whereas the children in the present study had received a full year of literacy instruction. This difference in the timing of measurement might also explain similar findings obtained by Silinskas et al. (2012). Further, Silinskas et al. (2020) found, with the full sample of about 2,000 Finnish families, that teaching reading at the end of kindergarten predicted early literacy skills (i.e., letter knowledge and reading skills) at the beginning of Grade 1. Therefore, it seems that a key factor to explain the discrepancy across studies seems to be the timing of measurement.

In terms of mothers’ responsiveness, we did find the expected shift from positive to negative correlations between the frequency of teaching and children’s literacy skills as children progressed from kindergarten to Grade 2 in the correlations (see Table 2) as well as the path model. Specifically, we found that children’s early literacy in kindergarten predicted negatively parental teaching at the end of Grade 1. The interpretation of this finding required subgroup analyses that clarified the pattern of teaching behaviors. As was the case in Sénéchal and LeFevre (2014), these analyses confirmed that mothers were responsive to their children’s developing literacy skills (see Figure 3A). Mothers of children who had the lowest level of word-reading skill at the end of Grade 2 increased the frequency of their teaching starting in Grade 1 whereas mothers of the children with the strongest skills decreased their teaching of reading. In contrast, mothers of children with reading closer to the sample average maintained the frequency of teaching across time. As such the present findings allow for a clear interpretation of similar findings obtained with the same dataset (Silinskas et al., 2012) and a different dataset (Silinskas et al., 2010a). It is not that parents are inefficient teachers, but rather, that parents are responsive to the pace of their children’s learning to read. The fact that a similar pattern was found in two different cultures and orthographies is remarkable.

### Child Interest

Across time points, children indicated that they were interested in letters and reading activities, with 74% of them choosing one of the two smiling faces. This is not to say, however, that children’s reports were highly stable. In fact, the inter-correlations among interest reports across time points ranged from 0.23 to 0.36. This latter finding suggest that, despite the skewness of the responses, there was more variability across time than the stability of the means indicated (Sperling and Head, 2002; Hume et al., 2015). Contrary to our predictions, child-reported interest was not correlated with other measures except for two modest coefficients with the mother-reported independent reading amount. However, the latter two coefficients were excluded from the path model because of the probability of chance findings. It is possible that during the early grades in Finland, when reading teaching is highly individualized, the children receive instruction that is well fitted to their individual levels and thus interest is retained even when reading skills are developing slower than average.

The absence of association between child interest and reading has been found in other recent reports (Kikas et al., 2015; Walgermo et al., 2018; Pezoa et al., 2019). In another report, the association was very modest and negative (e.g., *r* = −0.08, *p* = 0.05, *N* = 1,171, McTigue et al., 2019). In addition, lack of associations between child interest and reading skills might be a consequence of children’s overestimation of their own competences. For instance, in German studies, children in Grades 1 and 2 held overoptimistic self-concepts and, thus, high correlations with achievement were unlikely (Helmke, 1999). Finally, although small scale studies among preschoolers had found positive links between children’s degree of interest and skills on specific dimensions such as the alphabet (Frijters et al., 2000; Martini and Sénéchal, 2012), large scale studies among kindergarteners suggest that other aspects of motivation, such as task persistence might show stronger links to children’s reading skills (Kikas and Silinskas, 2016; Viljaranta et al., 2018).

### Limitations

There are limitations in the present study that require mention. First, maternal self-reports were used to assess home literacy practices and children’s independent reading. It is therefore possible that social desirability might be a factor in their answers. The distribution of responses as well as the lack of skewness suggest that, in this sample, there was variability in mothers’ responses. Although previous research has often used similar self-reports, future studies should consider observational measures (Tracey and Young, 2002), diary method (Pomerantz and Eaton, 2001) and in-depth interviews (Xu and Corno, 1998) with parents to confirm and expand the present findings. Second, maternal home literacy activities were measured by single items, which is not optimal. We asked mothers to report on teaching of reading as the key aspect of code-related activities, whereas other studies also included a wider range of items, such as teaching letters or writing (Aram and Levin, 2002; Reese et al., 2010). We asked mothers about the frequency of their shared reading as the key aspect of meaning-related home literacy activities, although more questions, for instance, about duration of shared reading, exposure to shared reading from other people could have been included (Scarborough and Dobrich, 1994). This being acknowledged, the present findings are consistent with previous studies that relied on multiple measures of each construct (e.g., Sénéchal and LeFevre, 2002, 2014).

### Practical Implications

The present study has some practical implications. First, parents should be encouraged to engage in literacy activities with their children at home. This engagement seems especially important in kindergarten, because not only was it related to growth in children’s skills from kindergarten to Grade 1, it was also related longitudinally to children’s reading on their own. Second, parents were responsive to children’s pace of reading acquisition, especially in Grade 1. Given this, teachers and other practitioners could use this opportunity to advise parents on the optimal ways of engaging in home literacy activities with their struggling children. Third, the results of the current study were based on the representative sample of Finnish families in terms of home language, SES, culture, and ethnicity. They also were based on children learning to read a transparent written language, and for whom Grade 1 begins at age 7. As such, they could be generalizable to families in other countries with relatively homogenous language environments and cultures (e.g., Silinskas et al., submitted). At the same time, it is important to note that similar findings to the ones presented here were obtained in opaque language environments, albeit appearing later than in Grade 1 (e.g., Sénéchal and LeFevre, 2014).

### Conclusion

This study documented the longitudinal interrelations among parental home literacy activities, children’s reading skills, interest, and their independent reading from kindergarten to Grade 2. This design allowed us to expand the Home Literacy Model (Sénéchal and LeFevre, 2002, 2014) in three ways. First, we demonstrated that both shared reading and parent teaching in addition to early literacy skills, all measured in kindergarten, predicted longitudinally Finnish children’s independent reading at the end of Grade 1. Second, children with stronger early literacy skills in kindergarten read independently more frequently once they were in Grade 1. Third, we showed that mothers quickly adapted their teaching behaviors to their children’s progress in reading. As such, the present findings add support for the often cited notion that parents are key partners in their young children’s education.

## Data Availability Statement

The data analyzed in this study are subject to the following licenses/restrictions: The dataset used in this manuscript was from the First Steps Study (www.jyu.fi/alkuportaat Director: professor M-KL, University of Jyväskylä, Finland, marja-kristiina.lerkkanen@jyu.fi). The information in this dataset is anonymized following the guidelines of the Finnish Social Science Data Archive. An experienced data manager of the project is responsible for data storage, management, and documentation. Therefore, we are confident that any risks related to data management are minimal. Requests to access the dataset can be made by emailing professor M-KL and the data manager Dr. Kenneth Eklund. Requests to access these datasets should be directed to Dr. Kenneth Eklund, kenneth.m.eklund@jyu.fi.

## Ethics Statement

The studies involving human participants were reviewed and approved by The Ethical Committee of University of Jyväskylä. Written informed consent to participate in this study was provided by the participants’ legal guardian/next of kin.

## Author Contributions

The data are archival and taken from a large-scale study conducted in Finland. GS, MS, MT, and M-KL contributed to the hypothesized model tested. GS conducted the analyses and wrote an initial draft of the manuscript. MS completed the draft, while the remaining authors provided feedback. All authors have read and approved the content of the submitted manuscript.

## Conflict of Interest

The authors declare that the research was conducted in the absence of any commercial or financial relationships that could be construed as a potential conflict of interest.
